# Zika virus infection disturbs development of human muscle progenitor cells

**DOI:** 10.3389/fcimb.2025.1638589

**Published:** 2026-01-30

**Authors:** Cássia Rocha, Daniella Arêas Mendes-da-Cruz, Elisa Negroni, Vincent Mouly, Ieda Pereira Ribeiro, Myrna Cristina Bonaldo, Wilson Savino, Vinicius Cotta-de-Almeida, Dumith Chequer Bou-Habib, Ingo Riederer

**Affiliations:** 1 Laboratory on Thymus Research, Oswaldo Cruz Institute, Oswaldo Cruz Foundation, Rio de Janeiro, Brazil; 2 National Institute of Science and Technology on Neuroimmunomodulation (INCT-NIM), Oswaldo Cruz Institute, Oswaldo Cruz Foundation, Rio de Janeiro, Brazil; 3 Research Network on Neuroinflammation, Oswaldo Cruz Institute, Oswaldo Cruz Foundation, Rio de Janeiro, Brazil; 4 Sorbonne Université, Inserm, Institut de Myologie, Centre de Recherche en Myologie, Paris, France; 5 Laboratory of Experimental Medicine and Health, Oswaldo Cruz Institute, Oswaldo Cruz Foundation, Rio de Janeiro, Brazil

**Keywords:** myoblasts infection, ZIKV, myotubes, myogenesis, cell migration, cell differentiation, cell proliferation

## Abstract

Zika virus (ZIKV) infection has emerged as a global public health emergency due to its expansion capacity and ability to cause neurological and congenital diseases. Muscle cells are targets for ZIKV, and myalgia and muscle disorders are frequently related symptoms during infection. We have previously demonstrated that myoblasts, the proliferating muscle stem cells essential for muscle repair, are permissive to ZIKV infection, generating infectious viral particles. In contrast, differentiated myotubes, derived from myoblast differentiation and fusion, control ZIKV replication. Nevertheless, little is known about the impact of ZIKV infection on muscle myogenesis. Using an *in vitro* model of skeletal muscle regeneration, human myoblasts were infected with the ZIKV-Rio-U1 strain, and their proliferation, adhesion, migration, and differentiation/fusion properties were analyzed 72 hours post-infection. We found that ZIKV replicates within myoblasts, promoting biological alterations such as the inhibition of cell cycle progression, preventing cell proliferation. Infected myoblasts exhibit poor adhesion, lack of membrane elongation, a reduced cell area, and decreased migratory capacity. Moreover, infection impaired the fusion of human myoblasts. Although differentiated and fused myotubes control ZIKV infection, proliferating infected myoblasts present an altered myogenic program. These results strongly suggest that ZIKV infection can affect myogenesis, modulating key biological processes crucial for skeletal muscle differentiation and regeneration. Accordingly, it is conceivable that ZIKV infection may impact myogenesis during embryogenesis, growth, and subsequent regenerative episodes during the adult period.

## 1 Introduction

The Zika virus (ZIKV) is an arbovirus transmitted to human hosts via arthropod vectors, predominantly Aedes spp. mosquitoes in tropical climates. ZIKV poses a global public health threat due to its rapid spread to various regions and countries, and also due to infection-associated complications (Metsky et al., 2017; Wang et al., 2017; Pielnaa et al., 2020). The first confirmed ZIKV infection in Brazil in 2015 triggered a rapid national outbreak, and by the beginning of 2016, the epidemic had spread explosively throughout the Americas and globally (Brasil et al., 2016; Lowe et al., 2018; Alaniz, 2019; Pierson and Diamond, 2020). Although the incidence of ZIKV disease has declined globally from 2017 onwards, transmission persists at low levels in several countries in endemic regions (World Health Organization, 2024). Due to climate change, the spread of ZIKV infection has increased in low-temperature and high-altitude regions. It is estimated that more than 100 million people worldwide will be in thermal conditions favorable to transmission over the next 50 years (Kraemer et al., 2019; Colón-González et al., 2021).

ZIKV is an arbovirus belonging to the Flaviviridae family and Flavivirus genus, which includes viruses such as Dengue (DENV), Yellow Fever (YFV), and West Nile (WNV) viruses (Neufeldt et al., 2018). The viral particle structure comprises a viral envelope composed of a lipid bilayer derived from the membrane of infected host cells and viral proteins. The viral envelope surrounds the icosahedral-shaped protein capsid, which anchors the genetic material, a single RNA strand with positive polarity. The 5′-terminal ends of the ZIKV genome carry a methylated nucleotide cap, which enables the translation of both structural and viral replication proteins (Heinz and Stiasny, 2017; Sirohi and Kuhn, 2017). Like other arboviruses, most ZIKV infections are asymptomatic or may manifest with mild “Dengue-like syndrome” symptoms, including fever, conjunctivitis, rash, arthralgia, and muscular symptoms such as myalgia (Brasil et al., 2016; Gorshkov et al., 2019). Nevertheless, neurological complications have been associated with ZIKV infection (Marques V de et al., 2019). ZIKV infection during pregnancy may cause congenital Zika syndrome (CZS), which is characterized by abnormalities, including microcephaly, arthrogryposis, hypertonia, and ocular, auditory, and musculoskeletal lesions, among others. CZS abnormalities can also occur in the postnatal period, causing microcephaly, neuromotor delay, and seizures in the first months of life (Lowe et al., 2018; De Paula Guimarães et al., 2019; Marques V de et al., 2019; Cavalcante et al., 2021). In addition, ZIKV infection has been correlated with an increase in the number of cases of Guillain-Barré Syndrome that affects primarily adults (de Oliveira et al., 2017; Nóbrega et al., 2018; Halani et al., 2021).

Skeletal muscle is the most abundant tissue in the body, with a remarkable capacity to regenerate after injury and insults (Tidball, 2017; Mukund and Subramaniam, 2020). Muscle tissue comprises post-mitotic, multinucleated, and contractile myofibers, which are formed through a process called myogenesis and a small population of stem cells known as satellite cells (SC) (Snijders et al., 2015; Li et al., 2025). Myogenesis occurs during both embryonic development and muscle growth in postnatal life. Asymmetric cell divisions maintain stem and progenitor cells essential for the specification of the myogenic lineage. Following activation, part of the SC population is expanded by cell proliferation, while another part returns to a quiescent state. The expanding population undergoes terminal differentiation and fuses to form myotubes, which ultimately will become mature fibers. This process occurs during both development and regeneration after skeletal muscle injury, which is of fundamental importance for maintaining muscle function and homeostasis (Schmidt et al., 2019; Gattazzo et al., 2020).

Myalgia is a frequently reported symptom in different viral infections and can persist beyond the acute phase of the infection, especially following flavivirus and alphavirus infections, as well as related to other viruses of clinical importance, such as Influenza and Coronavirus (Filippone et al., 2020; Ausems et al., 2021; Pitscheider et al., 2021; Appelman et al., 2024; da Silva et al., 2024; Study, 2024; Santiago et al., 2025). A study on ZIKV infection reported myalgia in 74% of infected patients (Bandeira et al., 2020), and ZIKV tropism for muscle tissues has been described in several studies (Filippone et al., 2020). In animal models, ZIKV infection causes muscle fiber degeneration, intense inflammatory infiltration, necrosis, and viral replication (Aliota et al., 2016; Gavino-Leopoldino et al., 2021). ZIKV tropism to muscle tissue has been reported during embryogenesis and infections after birth by maternal transmission (Gorshkov er al., 2019). The persistence of viral RNA was observed in muscle tissue up to 35 days after ZIKV infection in Rhesus monkeys (Hirsch et al., 2017). *In vitro* studies have demonstrated that human proliferating myoblasts are susceptible to ZIKV infection, allowing virus replication, whereas myotubes are resistant (Riederer et al., 2022; Legros et al., 2020). On the other hand, we observed that ZIKV invades the cell but does not progress to replication in 3-day differentiated human myotubes (Riederer et al., 2022). In this study, we identified antiviral genes and pathways that were overexpressed or only expressed in infected myotubes (Riederer et al., 2022).

Among the alterations in gene expression, ZIKV infection induces deregulation in the skeletal muscle regeneration process. Notably, ZIKV replication in the mouse skeletal muscle precedes the detection of viral RNA in the brain, and these infected muscle present necrotic areas, inflammation foci, and fiber atrophy (Gavino et al., 2021), suggesting that this tissue may be a site of viral reservoir. Herein, we aimed at analyzing the proliferation, adhesion, migration, differentiation and fusion of human muscle cells infected with ZIKV *in vitro*. These major biological processes related to myogenesis are fundamental for muscle formation, growth, repair and regeneration (Majchrzak et al., 2024; Wu and Yue, 2024).

## 2 Materials and methods

### 2.1 Cells

The KM155C25 human cell line, referred to as C25 throughout this paper, was kindly provided by MyoLine, the platform for immortalization of human cells of the Institute of Myology of Paris, France, coordinated by Dr. Anne Bigot. The myoblasts were isolated from the semitendinosus muscle of a healthy 25-year-old male donor and immortalized by the overexpression of telomerase hTERT and cyclin-dependent kinase 4, thereby extending their proliferative capacity (Thorley et al., 2016). Cells were maintained as myoblasts in proliferation medium (PM) composed of four parts DMEM High glucose (Gibco, #10566016) and one part of medium 199 (Gibco, #41150020), supplemented with 20% FBS (fetal Bovine Serum #SH3008403 Cytiva) and growth factors: dexamethasone (0.2 µg/mL Sigma #D4902), epidermal growth factor (EGF 5 ng/mL Gibco #PHG311), Fetuin (25 µg/mL Sigma Aldrich #341506), basic fibroblast growth factor (bFGF 0.5 ng/mL Gibco #PHG0026) and gentamicin (50 µg/mL Gibco #15750060). To induce myoblast differentiation, cells were seeded in PM until they reached high confluence (90%). Then, the medium was replaced with differentiation medium (DM), which consisted of DMEM high glucose supplemented with insulin (10 μg/mL, Sigma #91077C). The cells were cultivated at 37 °C and 5% CO^2^.

### 2.2 ZIKV infection

The ZIKV strain RIO-U1 (GenBank Accession number: KU926309) was isolated in 2016 from a urine sample of a patient from Rio de Janeiro, Brazil (Bonaldo et al., 2016). In this work, all experiments were performed at MOI 0.1, the same MOI used in our previous paper, where we compared gene expression profiling between myoblasts and myotubes (Riederer et al., 2022). Myoblasts were seeded in 100mm Petri dish at a density of 3.5x10^3^ cells/cm^2^ (Corning #430167) in PM. Aliquots of the virus were diluted at an MOI of 0.1 in 2 mL of PM without growth factors, supplemented with 5% FBS, and added to the cells for 2 hours (2 h) at 37 °C, 5% CO_2_. Non-absorbed viruses were removed, and 10 mL of PM supplemented with 20% FBS was added to the cells and cultured for 3 days. As a control, uninfected cells (MOCK) were grown under the same conditions described above.

### 2.3 Viral titration

Plaque assays were performed to determine viral titters (plaque-forming units, PFU). Vero E6 cell monolayers (5x10^4^/cm^2^) cultivated in 24-well plates (Corning) were incubated with 200μL of 10-fold serial dilutions of ZIKV for 2 h at 37 °C. After 1 h of incubation at 37 °C, the inoculum was removed and replaced with 1 mL of 2.4% carboxymethyl cellulose (CMC #C5013, Sigma-Aldrich) in Earle’s 199 medium (M0650, Merck). After 7 days of incubation at 37 °C, cells were fixed with 10% formaldehyde (P1005040000081, Dinamica) overnight, washed, and stained with 0.4% crystal violet (#C0775, Sigma-Aldrich) diluted in 20% methanol (34885, Sigma-Aldrich) to visualize plaques.

### 2.4 Viral replication inhibition assay

To determine the antiviral activity of the NITD008 inhibitor (SML2409, Sigma) in our model, 6x10^3^/cm^2^ human myoblasts were grown on coverslips (13 mm) in 24-well tissue culture and infected with MOI 0.1 as described. After 2 h of ZIKV incubation, the cultures were treated with NITD008 at the following concentrations: 1, 2, and 5 μM (diluted in PM). After 72 h post-infection (72 hpi), supernatants were collected, and viral titters were quantified by plaque assay in VERO cells in triplicate for each concentration. A vehicle-only with a 5 μM dimethyl sulfoxide (DMSO #20688, ThermoFisher Scientific) concentration was set as the control treatment. In addition, cytotoxicity was analyzed in the supernatants of ZIKV-infected myoblasts and MOCK, and an LDH cytotoxicity assay Kit (Lactate Dehydrogenase (LDH) Colorimetric Activity #EEA013, Invitrogen) was performed according to the manufacturer’s instructions.

### 2.5 Myoblast differentiation and fusion

The effects of ZIKV on myoblast differentiation were analyzed using two protocols. First, myoblasts were grown at a density of 1.2 × 10^4^ cells/cm^2^ in Lab-Tek 8-well Permanox cell culture slides (#177445, Thermo Scientific) in PM (20% FBS and growth factors) and infected with ZIKV at MOI 0.1 (100 µL/well of medium DMEM and 199 (1:5) supplemented with 5% FBS), after 2 h incubation, the cultures were then treated with NITD008 5 µM in PM for 72 h. And then, the PM was changed to DM for an additional 72 h to induce cell differentiation. In the second protocol, myoblasts were seeded at a high confluence, 7.5x10^4^ cells/cm^2^ in Lab-Tek 8 well cell culture slides and infected with ZIKV. After a 2 h incubation and washing, DM was added to the cells, which were then cultured for an additional 72 h. A fusion index (FI) was calculated to evaluate the ability of myoblasts to differentiate into myotubes. The percentage of nuclei inside myotubes (cells with at least two nuclei) was determined over the total number of nuclei. Myocytes were defined as mononuclear and differentiated cells (positive for MF20). Cell cultures were stained with the anti-myosin heavy chain (MyHC) antibody (MF20 clone) by immunofluorescence to identify differentiated and fused myoblasts, as described below.

### 2.6 Immunofluorescence

After 72 hpi, myoblasts were fixed with 4% PFA (Paraformaldehyde, Sigma Aldrich #158127) for 10 minutes (min), then permeabilized with 0.3% Triton X-100 (#42K0217 Sigma) in PBS for 15 min at room temperature. Fixed cells were blocked in buffer containing 1% bovine serum albumin (BSA #A0296), 2% FBS (Gibco # 12657029), and 0.1% Triton X-100 in 1x phosphate buffer saline (PBS Gibco#15374875) for 40 min. The cells were incubated for 1 h with anti-E Flavivirus protein antibody 4G2 (140719S0004G2P-Bio-Manguinhos, 1:50) or anti-desmin rabbit (AB907 Sigma-Aldrich, 1:30), followed by incubation with a fluorophore-conjugated secondary antibody (1:200) diluted in blocking buffer: anti-mouse Alexa Fluor 546 (A- 11030 Invitrogen) or anti-rabbit Alexa Fluor 488 (A-21206 Invitrogen) for 30 min in the dark. For myoblast differentiation and fusion staining, cells were incubated for 1 h with an anti-MyHc antibody (AB 2147781 Hybridoma Bank, MF20 clone 1:30), followed by incubation with anti-mouse Alexa Fluor 546. Nuclei were stained with DAPI (4′,6-diamidino-2-phenylindole) diluted in H2O at 1:20,000 (Life Technologies, D1306) for 10 min in the dark. The slides were mounted using Prolong Gold Antifade Mounting Media (P36930 Invitrogen). All incubations were performed at room temperature in the dark. Images were acquired using an AxioImager A2 fluorescence microscope with AxioVision Rel4.8 software (Zeiss, Oberkochen, Germany) or a Leica TSC SP8 Confocal device and LAS-X Software (Leica Microsystems, Wetzlar, Germany).

### 2.7 Morphology assay

To quantify the number of nuclei and morphological changes, image analysis was performed using the software ImageJ FIJI (https://imagej.net/tutorials/). The “set scale” tool was used to measure distances in micrometers (µm). For initial image preparation, the “Image, Color, Split channels” function was applied, and channel AF488 was selected, which defines the structure being studied (desmin, a cytoskeletal protein in muscle cells). Threshold tool to reduce noise and “Binary, fill holes” to fill empty spaces, which improves the visibility of structures. “Analyze > Analyze Particles” function allows quantification of parameters such as cell area, circularity, roundness, and perimeter to analyze structure. Cells at the edge were defined as an exclusion criterion. For that, images were acquired using an Axio Imager A2 fluorescence microscope (Zeiss).

### 2.8 EdU assay proliferation

Cells in proliferation were detected using EdU (5-ethynyl-2′- deoxyuridine), a thymidine analog (EdU- Click 488 #FG07008), according to the manufacturer’s instructions. Infected cells were grown on coverslips (13 mm) for 72 h in PM. Over the last 2 h, cells were incubated with EdU at a final concentration of 5 µM in 300 µL of PM without growth factors at 37 °C and 5% CO^2^. 72 hpi, cells were washed with PBS, fixed with 4% PFA for 10 min, and permeabilized with 0.5% Triton X-100 (42K0217, Sigma) in PBS for 20 min at room temperature. After a washing step with 3% BSA in PBS, cells were stained with EdU buffer detection for 30 min in the dark, using the components diluted according to the manufacturer’s instructions. After EdU staining, immunofluorescence was performed to detect ZIKV E protein (protocol described above). Images were acquired using an AxioImager A2 fluorescence microscope with Axio Vision Rel 4.8 software (Zeiss, Oberkochen, Germany). Quantification was performed using the FIJI ImageJ software.

### 2.9 Flow cytometry

To analyze the expression of adhesion molecules and the presence of viral proteins by flow cytometry, 3,5x10^3^/cm^2^ myoblasts were seeded in 100 mm Petri dishes, infected with ZIKV, and cultured for 72 h. Myoblasts were trypsinized for 5 min at 37 °C (Trypsin/EDTA 0.25% Invitrogen) to remove adherent cells, then washed with PBS and centrifuged at 400g at 4 °C for 5 min. To detect intracellular molecules, cells were fixed with 4% PFA for 20 min at room temperature. After washing with PBS and centrifugation (400g, 5 min at 4 °C), cells were permeabilized using a Perm Buffer permeabilization/blocker buffer containing 0,3% Triton, 8% human serum (H4522 Sigma) in PBS for 1 h at 4 °C. After centrifugation at (400g, 5 min 4 °C) to remove the excess of the perm buffer, cells were incubated with the primary antibody 4G2 (Bio- Manguinhos, Fiocruz 1:200) for 1 h at 4 °C followed by incubation with an anti-mouse AF546-conjugated secondary antibody (#A11003 Invitrogen 1:300) for 30 min or anti-ki67 BV421 (#562899 Clone B56 BD Biosciences) at 4 °C. Cells were washed twice in PBS, and samples were acquired.

### 2.10 Apoptosis assay

After 72 hpi, ZIKV-myoblasts and MOCK were washed with PBS, centrifuged at 400G, 4 °C, 5min, and resuspended in 300µL of 1x annexin-V binding Buffer (#556454, BD Bioscience) diluted in H_2_O. Then, cells were incubated with 5 µL of Annexin-V APC (550475, BD Biosciences) for 15 min at room temperature. PI (6 µg/mL) was added to the cell suspension at room temperature for 2 min prior to acquiring samples in flow cytometry Experiments were performed using a FACS Celesta II cytometer (BD Biosciences) and analyzed with FlowJo software (V10).

### 2.11 Cell viability

To determine the viability of myoblasts, cells removed by trypsinization after 72 and 120 hpi were incubated with FVS520 cell viability dye (564407, BD Biosciences) diluted in PBS (1:4000) for 15 min at 37 °C. Positive controls (dead cells) were obtained by incubation with 70% ethanol for 3 min. For this analysis, live and dead cells were acquired using a FACS Celesta II cytometer (BD Biosciences) and analyzed with FlowJo software (V10).

### 2.12 Cell cycle

DNA content was analyzed using flow cytometry to estimate the percentage of the cell population within each cell cycle phase. 72 hpi, cells were removed by trypsinization, washed with PBS, centrifuged at 400g, 4 °C for 5 min, and fixed with ice- cold 70% ethanol (diluted in distilled water) at 4 °C for 30 min. Cells were then washed with PBS and incubated with a hypotonic solution containing 50 µg/mL Propidium Iodide (#P3566–1 mg/mL Invitrogen), 100 µg/mL Ribonuclease A (RNAse A # 12091021), 2 mM MgCl2 (#208337 Sigma Aldrich), diluted in PBS for 40 min at room temperature in the dark. The samples were acquired using the FACS Celesta II cytometer (BD Biosciences) and analyzed using FlowJo software (V10).

### 2.13 Number of cell divisions

Myoblasts were infected under the conditions described above. At 72 hpi, ZIKV-myoblasts and MOCK were submitted for counting the number of cell divisions. The formula used for calculating the number of cell divisions is derived from the basic principle of exponential growth (Wang et al., 2015). They enable us to estimate the number of cell divisions that occurred during the observed growth period or the average number of complete mitoses per cell during that period. *n* is the division number, *n*0 represents the initial number of cells (plated cells number), *n*t represents the final number of cells (counting day), as described below:


$$n=\frac{\log(n_{o}/n_{t}}{\log2}$$

### 2.14 Cell adhesion assay

The adhesion capacity of ZIKV-infected myoblasts was estimated after 72 hpi culture period in PM. 4x10^3^ cells/cm^2^ were seeded (in 100 µL of DMEM without serum) on coverslips (18 mm), pre-coated with ECM (extracellular matrix) protein: human recombinant Laminin isoform 111 (LM-111) 5 µg/mL (#LN111–02 BioLamina) or without coating for 2 h. LM-111 was diluted in PBS with calcium and Magnesium (#14040141 Gibco) and incubated for 1 h after adhesion at 37 °C, 5% CO2. LM-111 excess was removed, and myoblasts were allowed to adhere for 2 h at 37 °C. Non-adherent cells were gently removed by two washes with PBS, and the adherent ones were fixed with 4% PFA for 10 min and permeabilized with 0.3% Triton X-100 for 15 min. A blocking buffer containing 1% BSA, 2% SFB, and 0.3% Triton X-100 in PBS was added to the cells for 40 min. The desmin and ZIKV protein E detection protocol was performed using the immunofluorescence protocol above.

### 2.15 Migration assay

A Transwell migration assay was performed to evaluate the migratory capacity of human myoblasts that had been grown in 100 mm Petri dishes for 72 hpi. A cell suspension was seeded in membrane inserts on a 24-well plate using membranes with 8-μm pore sizes (Corning #3422), which were pre-coated with LM-111 at 5 μg/mL for 1 h at 37 °C and 5% CO^2^. Infected and MOCK myoblasts (72 hpi) were seeded (5x10^4^/cm^2^) in the upper chamber in 100 μl of DMEM without serum, and 600 μl of the migration medium containing DMEM supplemented 1% FBS was put in the lower chamber as a chemoattractant. Cells were allowed to migrate for 4 h. Inserts were washed with PBS, and cells that did not migrate through the porous membrane were gently removed from the upper side of the insert with a cotton swab. Cells that migrated to the lower chamber were fixed with 3.7% formaldehyde for 15 min, washed with PBS, and stained with 0.4% crystal violet (42555 Sigma) diluted in 20% methanol for 10 min. Inserts were air-dried and counted under an optical microscope at 200x magnification. At least ten random microscopic fields were counted in each insert.

### 2.16 Statistical analysis

Data are presented as mean ± SD, the p values represent significant differences detected, and p<0.05 were considered statistically significant (p<0.05). Normality tests were performed on all data, including the Shapiro-Wilk test, and the T-test, followed by the nonparametric Mann-Whitney test correction test, which was used for pairwise comparisons depending on the data distribution. Two way-ANOVA was used to compare more than three groups, or Kruskal-Wallis test, depending on the data distribution. The reported sample size for a given dependent measure represents the number of replicates per group in 3 or 4 separate experiments. The specific statistical tests used for each experiment are provided in the figure legends. Statistical analyses were performed using GraphPad Prism 10 software.

## 3 Results

### 3.1 ZIKV infection leads to a reduction in the number of human myoblasts

**Figure 1:**
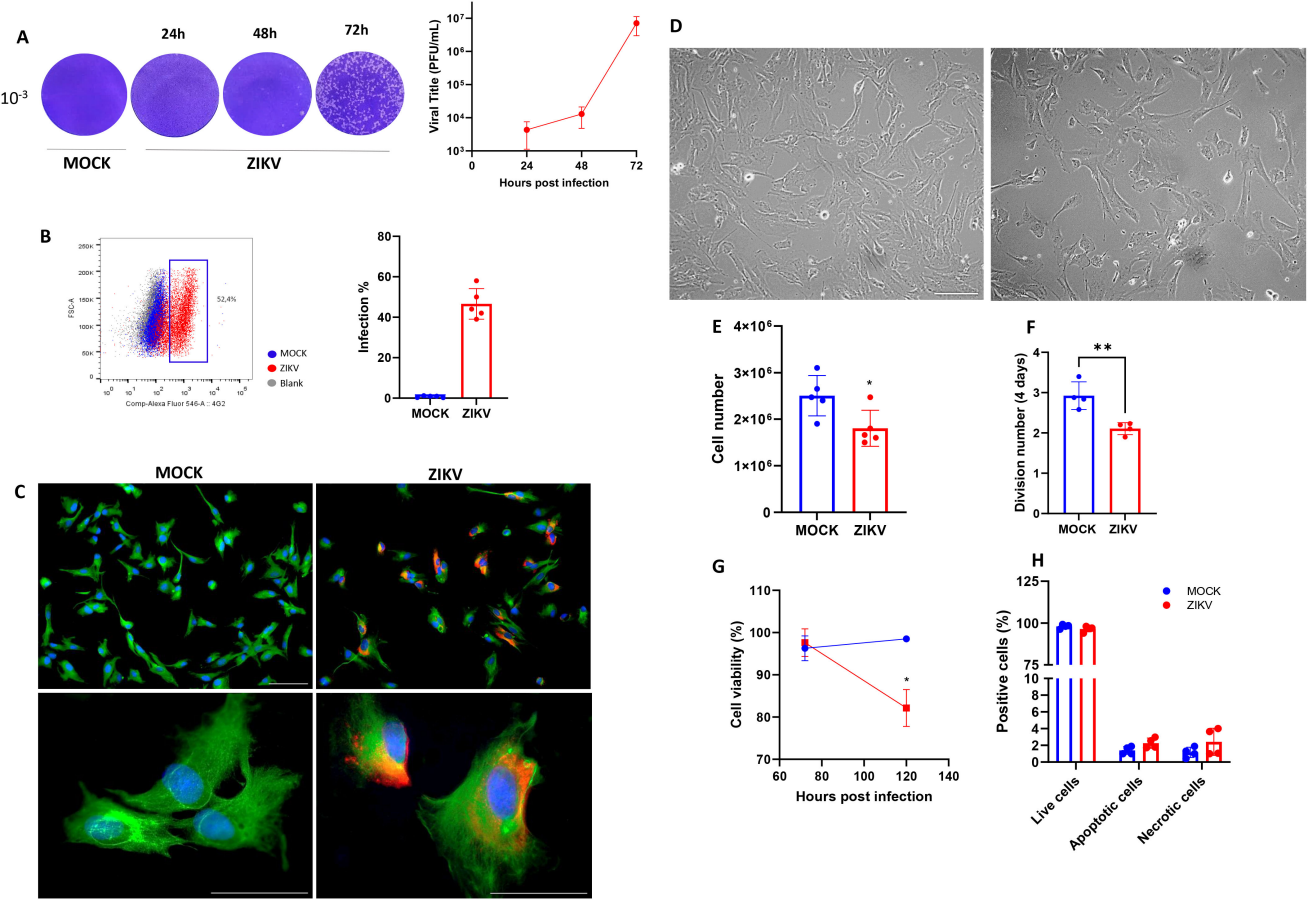
ZIKV infection in human myoblasts is productive and reduces the number of myoblasts in culture. **(A)** Representative images of ZIKV-infected (ZIKV) and mock- infected (MOCK) Vero cells. The graph shows the quantification of viral titters during the kinetics of infection (MOI 0.1). Each time point represents the mean ± SD of triplicate measurements from at least 3 independent experiments. **(B)** A representative dot plot of flow cytometry data showing the percentage of ZIKV-positive cells (4G2 labeling) in ZIKV (red) and MOCK (blue) infected cultures at 72 hpi, (Blank control for negative labeling in gray). The bar graph shows the percentage of ZIKV infection, as defined by the frequency of 4G2+ myoblasts, in infected (ZIKV; red) and mock-infected (MOCK; blue) cultures 72 hpi. Data are represented as mean ± SD of five independent experiments. **(C)** Representative immunofluorescence micrographs demonstrating the detection of the viral envelope E protein via 4G2 staining (red), desmin (green), and nuclei with DAPI staining (blue) in myoblast cultures 72 hpi. Scale bar 100 µM. The lower panels show ZIKV- and MOCK-infected myoblasts at higher magnification. Scale bar 50 µM. **(D)** Representative images of phase contrast microscopy of myoblast cultures at 72 hpi. MOCK-infected myoblasts on the left and ZIKV-infected myoblasts on the right. Scale bar: 100 µm. **(E)** The Graph shows the number of myoblasts in culture at 72 hpi. Statistical difference *p<0.05 using Unpaired T-test. Data are represented as mean ± SD of five independent experiments. **(F)** The Graph shows the number of myoblast divisions per day calculated using the exponential growth formula. Statistical difference ***p<0.001 using Unpaired T-test. Data are represented as mean ± SD of four independent experiments. **(G)** The Graph shows the percentage of viable cells, as determined by flow cytometry analysis using the FVS520 marker, in ZIKV-infected cultures compared to control MOCK cultures. Statistical difference *p<0.05 using Two-way ANOVA. Data are represented as mean ± SD of three independent experiments. **(H)** The Graph shows the percentage of live cells (annexinV-/PI-), apoptotic cells (annexinV+/PI-), and necrotic cells (annexinV+/PI+) detected by flow cytometry 72 hpi. Each time point represents the mean ± SD (n = 4). No statistical differences using Two-way ANOVA. Data are represented as mean ± SD of four independent experiments.

We first evaluated the potential cytopathic effect of ZIKV infection in progenitor muscle cells. Viral particle production was quantified by collecting supernatants from ZIKV- infected myoblasts (MOI 0.1) at 24, 48, and 72 hpi, and viral titters were determined using a plaque assay. **Figure 1** shows a progressive increase in viral titters up to 72 h (**Figure 1A**). Flow cytometry detection of the viral E envelope protein (using 4G2 antibody) indicated that approximately 50% of myoblasts were infected (**Figure 1B**). **Supplementary Figure 1** outlines the gating strategies. These results were confirmed by immunofluorescence (**Figure 1C**). At 72 hpi, lower cell density was observed in ZIKV myoblasts, and no morphological changes were observed in the cultures analyzed under phase contrast optical microscopy (**Figure 1D**). Infected cultures had a reduced number of myoblasts compared to MOCK controls (**Figure 1E**) and were not related to loss of cell viability at this time point; however, this reduction occurred later, at 120 hpi (**Figure 1G**). Moreover, no apoptosis was observed in human ZIKV-infected myoblasts compared to MOCK controls at 72 hpi (**Figure 1H**). Therefore, at 72 hpi, a reduction in the number of cell divisions was observed in ZIKV-infected myoblasts (**Figure 1F**), which could explain the decreased number of myoblasts in the infected cultures.

### 3.2 ZIKV infection disrupts the cell cycle in myoblasts

**Figure 2:**
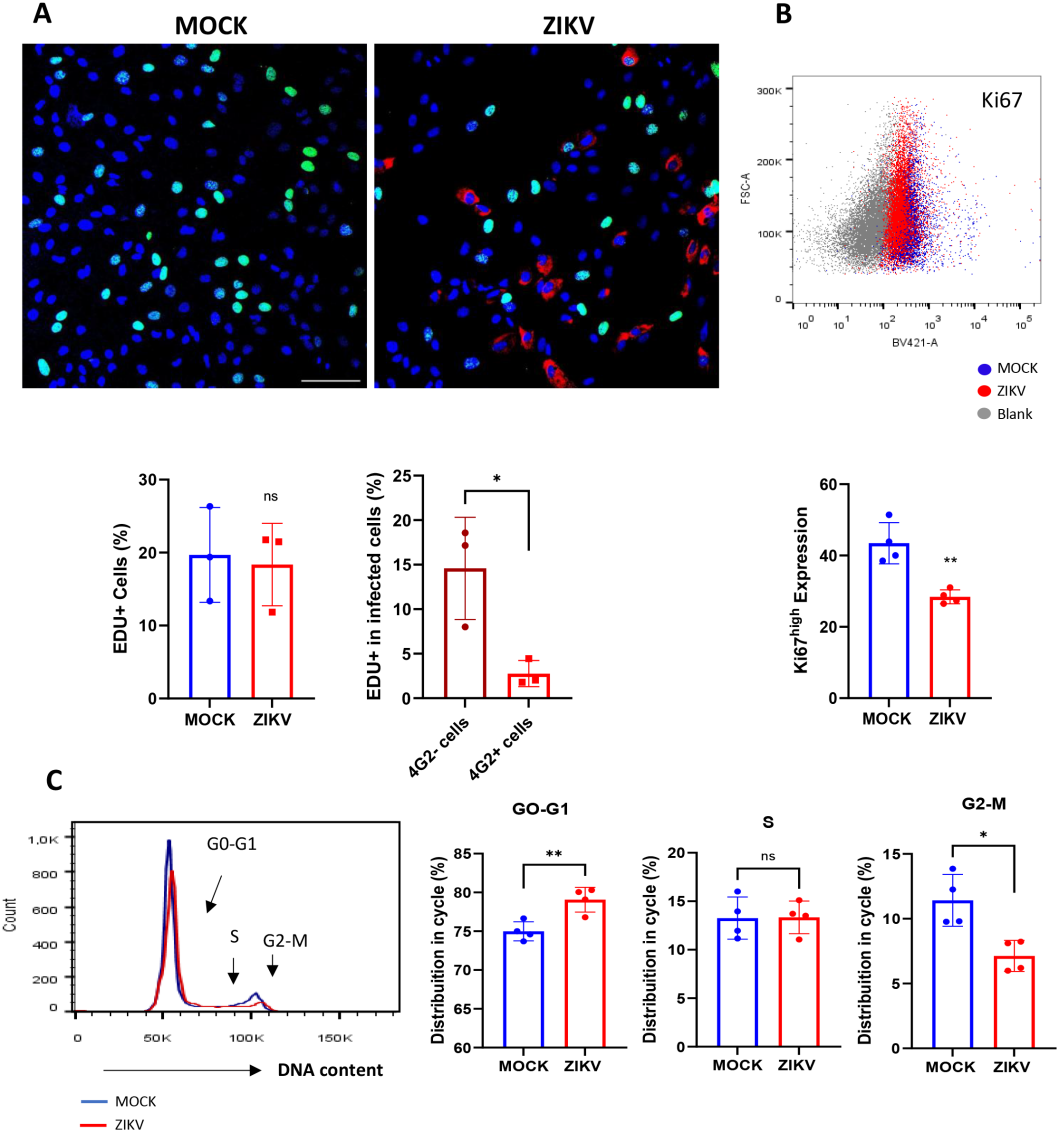
ZIKV infection disrupts the cell cycle in human myoblasts. **(A)** Representative immunofluorescence micrographs demonstrating EdU incorporation (green) and viral envelope detection with 4G2 staining (red), and nuclei stained with DAPI (blue) at 72 hpi. Scale bar 100 µm. The bar graphs below show the percentage of human myoblasts in proliferation (EdU+) at 72 hpi in MOCK- and ZIKV-infected cultures (left), and the bar graph in the right shows EDU+ cells within the infected culture (4G2+ and 4G2- myoblasts). Data is the mean ± SD of three independent experiments in duplicate. ns: no statistical difference, *p<0.05 using Unpaired T-test. **(B)** A representative dot plot of flow cytometry data showing the expression of KI67 in ZIKV (red - 4G2+ myoblasts) and MOCK (blue - 4G2- myoblasts) infected cultures at 72 hpi (Blank control for negative labeling in gray). The bar graph shows the percentage of myoblasts expressing KI67 at high density in the membrane (ki67^high^) in MOCK- and ZIKV-infected cultures at 72 hpi. Data are represented as mean ± SD of three independent experiments. Statistical difference **p<0.01 using an Unpaired T-test. **(C)** A representative histogram flow cytometry showing DNA content (propidium iodide, PI) in ZIKV (red) and MOCK (blue) infected cultures. The bar graphs show the quantification of percentual distributions of cells in the cell cycle phases: G0-G1, S, and G2-M. Statistical difference and **p<0,001 and *p<0.05, respectively, using Unpaired T-tests followed Welch’s correction. ns: no statistical difference. Data represents the mean ± SD of four independent experiments in duplicate.

Upon activation, muscle progenitor cells undergo multiple rounds of division, generating a high number of myoblasts, necessary for muscle regeneration (Dayanidhi and Lieber, 2014; Hindi and Millay, 2022). We evaluated whether ZIKV infection might impact myoblast proliferation using a nucleoside analog, EdU, which incorporates EdU during DNA synthesis. We observed no difference in the number of EdU-positive cells in ZIKV-infected cultures at 72 hpi compared to MOCK controls (**Figure 2A**). However, we found that infected myoblasts (4G2+ cells, immunolabeled for the E viral envelope protein) were almost all EdU negative (**Figure 2A**). We also observed a significant reduction in Ki67high infected myoblasts (**Figure 2B**). Ki67 is a nuclear protein expressed in cells during the cell cycle, most intensely in the S phase and mitosis (Dzulkifli et al., 2018). ZIKV-induced cell cycle arrest was confirmed in infected myoblasts 72 hpi by quantifying DNA content using PI, a DNA intercalator, via flow cytometry. Myoblasts decreased within the G2-M phase in the infected cultures, while an increase in the G0-G1 phases was observed (**Figure 2C**). These results may explain the lower number of cell divisions and consequently reduced cell numbers in ZIKV-infected myoblast cultures compared to MOCK controls (**Figures 1E, F**). However, our data does not answer whether ZIKV reduces, retains, or halts myoblast proliferation, and a more detailed evaluation of cell cycle pathways is needed to elucidate how ZIKV prevents myoblast proliferation.

### 3.3 ZIKV infection reduces myoblast adhesion capacity

**Figure 3:**
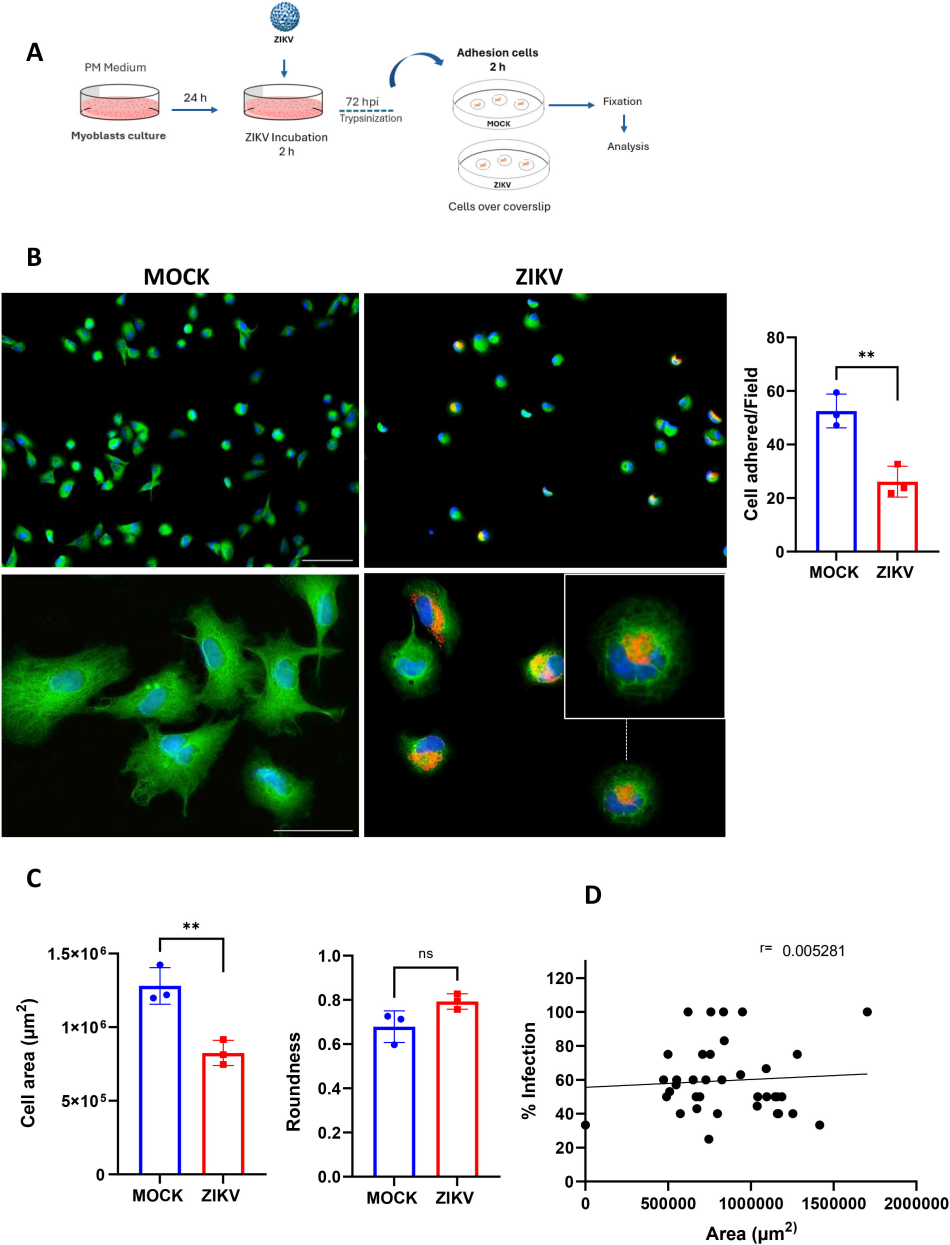
ZIKV infection reduces myoblast adhesion capacity. **(A)** A representative scheme of adhesion assay. At 72 hpi (MOI 0.1), ZIKV and MOCK cultures were trypsinized and transferred to glass coverslips to adhere for 2 h Adherent myoblasts were quantified and phenotyped using ImageJ software. The scheme was created using BioRender (BioRender.com). **(B)** Representative images of adhered cells stained for desmin (green), 4G2 E viral protein (red), and nuclei (DAPI). The Lower panels are myoblasts in higher magnification. Scale bar: 100 µm. The bar graphs on the right show the average number of adhered ZIKV- and MOCK-infected myoblasts per field after 2 h of incubation. 12 fields per coverslip were counted from three independent experiments. Statistical significance **p<0.01 using Unpaired T-tests followed Welch’s correction. **(C)** The bar graphs show the quantification of the average cell area (**p<0.01) and roundness (ns) of MOCK- and ZIKV-infected myoblasts per field at 72 hpi. Statistical difference using Unpaired t test with Welch’s correction, ns: no statistical difference. **(D)** Pearson’s correlation shows no significant differences in cell area between infected and non-infected cells within the ZIKV-infected myoblast cultures (r = 0.00, p< 0.6691). The y-axis represents the percentage of infection (infected myoblasts, 4G2+), and the x-axis represents the average cell area per well. Data are expressed as mean ± SD of the 11 fields counted from three independent experiments; each performed in duplicate.

Cell adhesion stimulates signals that regulate differentiation, migration, and survival. The first step of myoblast fusion is cell-to-cell recognition and contact, which will involve adhesion (Taylor et al., 2022). We investigated the adhesion capacity of ZIKV-infected myoblasts, and after 72hpi, the cells were detached from the cultures, seeded onto coverslips, and analyzed 2h after adhesion (**Figure 3A**). We observed that infected myoblasts exhibited lower adhesion than controls (**Figure 3B**). The cell area was significantly smaller, with less spread out in ZIKV-infected myoblasts than in controls, while circularity remained unchanged between groups (**Figure 3C**). These morphological changes were observed in all infected cultures, including those cells that were negative for ZIKV E viral protein labeling within the population (**Figure 3C**). Moreover, no significant correlation was observed between morphological alterations and the ZIKV viral E protein in infected (positive for ZIKV viral E protein) and non-infected cells (**Figure 3D**). The same effect was observed in wells were pre-coated with LM-111, an isoform known to stimulate myoblast adhesion and migration (González et al., 2017; Taylor et al., 2022; Dohi et al., 2025) (**Supplementary Figure 4**). Together, these results suggest that ZIKV infection reduces the ability of myoblasts to adhere and spread, an effect observed in both infected and non-infected cells within the same culture, likely due to a bystander effect.

### 3.4 ZIKV infection disrupts myoblast migration

**Figure 4:**
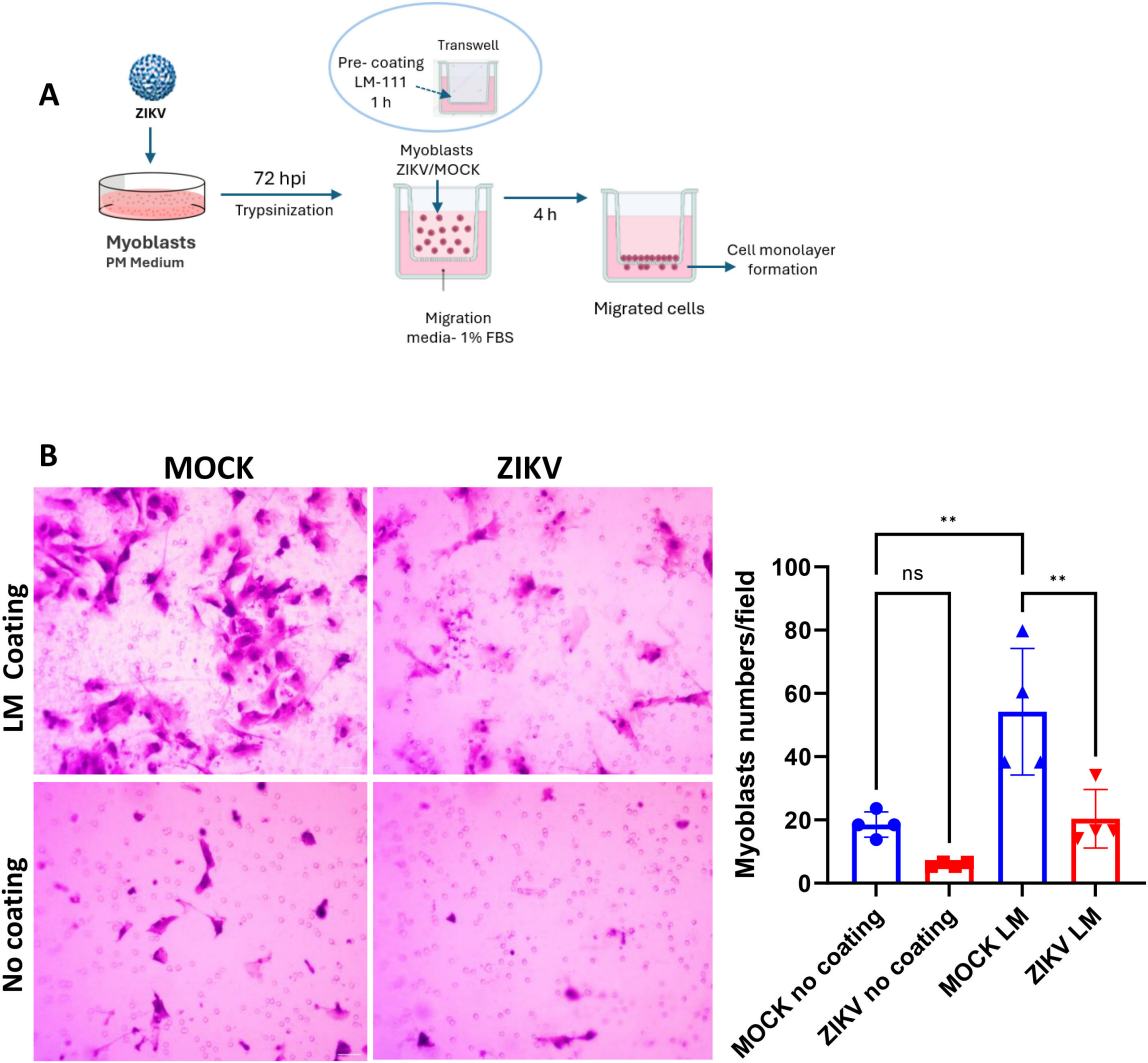
ZIKV infection disrupts myoblast migration**(A)** A representative scheme of the transwell transmigration assay with LM-111 (haptoptactic stimulus) and FBS 1% (chemotactic stimulus) in MOCK- and ZIKV-infected cells. At 72 hpi in culture, ZIKV-infected and MOCK myoblasts were allowed to migrate in Transwell chambers with 8 μM membrane pore sizes for 4 h Inserts were previously coated with laminin-111 (LM-111) or without coating, and the lower chambers were filled with FBS 1%. The scheme was created using BioRender (BioRender.com). **(B)** Representative images of migrated myoblasts. Adhered cells that migrated to the other side of the transmembrane were stained with crystal violet, and images were acquired using an optical microscope scale bar 50 µm. The bar graph represents the mean ± SD of migrated myoblasts per field in four independent experiments, each performed in duplicate. Statistical significance **p<0.01 using Two-way ANOVA followed by Tukey’s multiple comparisons test. Data are expressed as mean ± SD of the fields, which is counted 10 fields from four independent experiments, each performed in duplicate.

Given that myoblasts migrate towards neighboring myoblasts or damaged fibers before fusing to form or repair myofibers (Hindi et al., 2017; Gao et al., 2024), we investigated the migratory capacity of myoblasts after ZIKV infection. To this end, Transwell migration assays were performed. **Figure 4A** shows the scheme of the transwell culture system. Infected myoblasts cultured for 72 hours were allowed to migrate from the upper chamber to the lower chamber through an 8 µm pore size membrane filter, pre-coated with LM-111 or without coating. Corroborating our previous results (Silva-Barbosa et al., 2008; González et al., 2017), LM-111 increased myoblast migration (MOCK LM) compared to the control without coating (MOCK no coating). However, ZIKV infection strongly reduced the migration capacity of myoblasts towards LM-111 compared to the LM-111 MOCK (**Figure 4B**). Taken together, these results suggest that ZIKV disrupts myoblast migration.

### 3.5 ZIKV infection inhibits myoblast fusion

**Figure 5:**
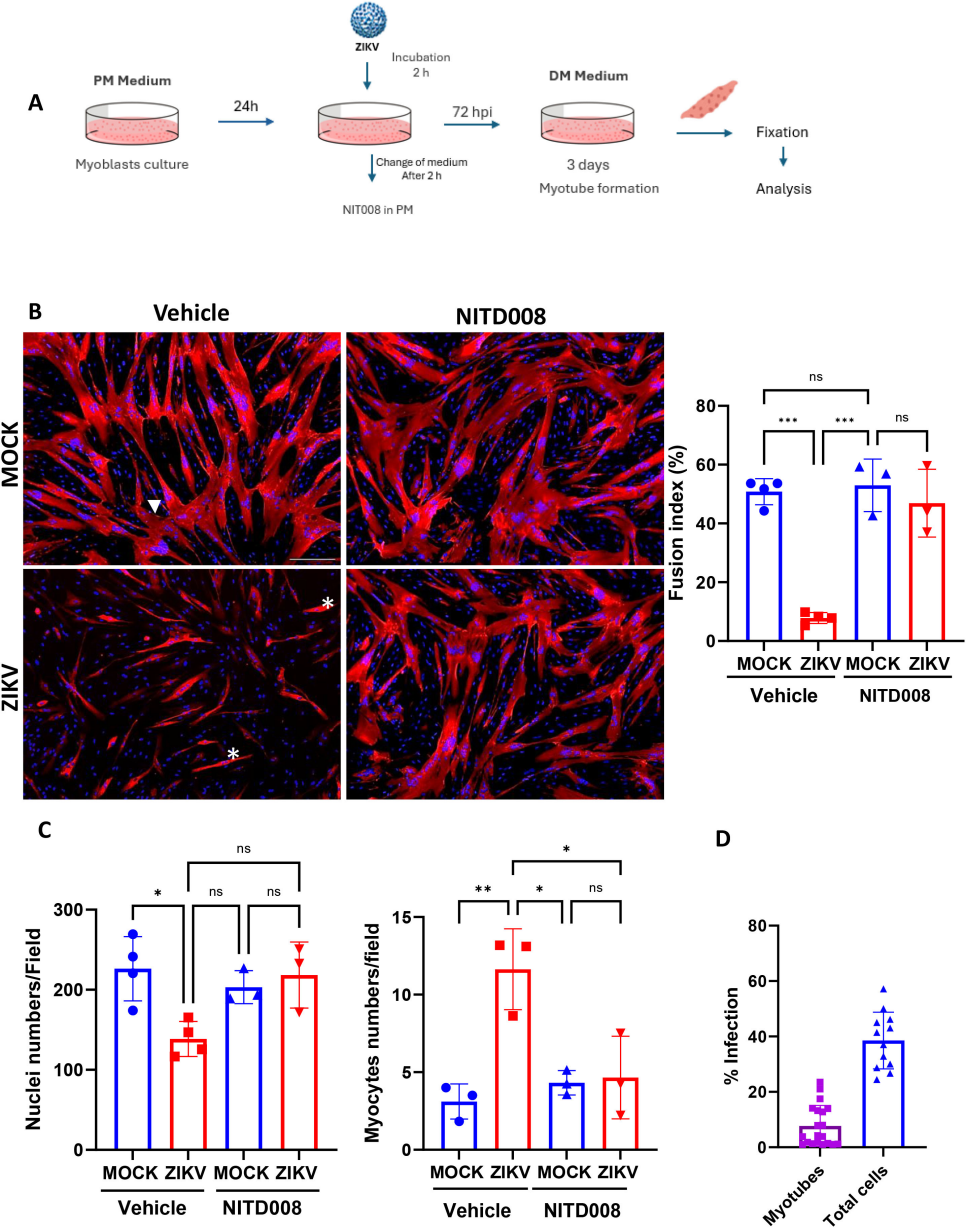
ZIKV infection inhibits myoblast fusion. **(A)** A representative scheme of differentiation assay. Myoblasts were infected (2 h) with ZIKV (MOI 0.1) and then ZIKV and MOCK-infected myoblasts were treated with NITD008 (or DMSO as vehicle control) in PM for additional 2 h to inhibit viral replication, and then cultivated in PM. After 72 hpi, the cells were allowed to differentiate for 3 days in DM. The differentiation and fusion indexes of ZIKV and MOCK-infected myoblasts, treated or not with viral inhibitor, were analyzed by immunofluorescence using the MyHC marker. **(B)** Representative immunofluorescence micrographs showing the formation of myotubes from ZIKV and MOCK. The differentiated cells were labelled with anti-MyHC antibody (red) and the nuclei with DAPI (blue). Asteristic shows mononuclear cells and arrowhead shows multinuclead fused cells. Scale bar 100 µm. The Bar graphs on the right show the fusion index of MOCK and ZIKV-infected cultures. The fusion index was calculated by the ratio of the number of nuclei within fused myotubes (MyHC+ cells containing 2 or more nuclei) versus the total number of nuclei. Statistical differences ***p<0.001 **p<0.01 and *p<0.05 using Two Way-ANOVA followed Turkey’s Test. ns: no statistical difference. **(C)** The graphic on the left shows the number of nuclei per field, and the graphic on the right shows the number of myocytes per field. Myocytes are defined as differentiated (MyHC+) and mononuclear (non-fused) cells. Statistical differences *p<0.05 **p<0.01 using Two Way-ANOVA followed the Turkey-test test. ns: no statistical difference. **(D)** Quantification of infection (4G2+ cells) myocytes and myotubes. The data are represented by the average of the fields counted (at least 10 fields) from three independent experiments, each performed in triplicate.

Myoblasts fuse into multinucleated syncytia, forming myotubes (Demonbreun et al., 2015). Myoblasts were maintained in PM for 72 hpi, then PM was replaced by DM to induce differentiation for an additional 72 h (**Figure 5A**). We observed that after 3 days of differentiation, ZIKV infection reduced the MyHC-positive population and myotube formation (**Figure 5B**). In addition, ZIKV infection reduced the total number of nuclei in myotubes, i.e., those containing at least 2 myonuclei formed from infected myoblasts, and increased the number of myocytes, defined as mononuclear cells expressing differentiation markers such as MyHC (**Figure 5C**). In brief, ZIKV infection decreases the total number of cells expressing MyHC. Actually, it reduces the number of fused cells, suggesting that ZIKV has a more substantial inhibitory effect on cell fusion than on cell differentiation. To further investigate the mechanisms underlying the impact of ZIKV on myoblast fusion, the replication inhibitor NITD008 was added to the cultures after incubation with ZIKV. NITD008 is a potent antiviral against ZIKV and other flaviviruses (Deng et al., 2016). Antiviral activity of NITD008 was confirmed in myoblasts infected with ZIKV (**Supplementary Figure 5**). Interestingly, we observed that inhibiting ZIKV replication restored myotube differentiation and fusion, with no alteration in cell numbers (**Figures 5B,C**). Alternatively, myoblasts seeded in high confluence (ready to differentiate) were infected, and the differentiation medium was added to the cells. With this protocol, differentiation was also affected, but to a lesser extent, compared to the differentiation of 3-day-infected myoblasts (**Supplementary Figure 6**). The viral protein was mainly detected in myocytes and was less detected in myotubes (**Figure 5D**). Conjointly, these results suggest that the effects of ZIKV infection on cell fusion may depend on viral replication, rather than just the presence of the virus.

## 4 Discussion

Myalgia, muscle weakness, myositis, rhabdomyolysis, atrophy, are frequently associated with arboviruses, among which DENV, CHIKV, ZIKV, WNV, as well as other infections of clinical importance, such as Influenza and SARS-CoV-2 (Ibrahim et al., 2018; Filippone et al., 2020; Legros et al., 2020; Lentscher et al., 2020; Pitscheider et al., 2021; Appelman et al., 2024; da Silva et al., 2024). Skeletal muscle tissue symptoms are particularly central in CHIKV and DENV infections, playing a crucial role in the pathogenesis of these diseases. CHIKV infection is known for its major effect on skeletal muscle function and quality of life, particularly during the chronic phase of infection (Lentscher et al., 2020; da Silva et al., 2024), when muscular symptoms can persist for months (Filippone et al., 2020). ZIKV has a broad capacity to infect different tissues, including skin, placenta, body fluids, brain, and thymus (Hamel et al., 2015; Woo et al., 2016; Aagaard et al., 2017; Hirsch et al., 2017; Miner and Diamond, 2017; Khaiboullina et al., 2019; Messias et al., 2020; Vota et al., 2021; Rychlowska et al., 2022), and infection of skeletal muscle cells was confirmed both *in vitro* and *in vivo* in experimental models (Gavino et al., 2021; Aliota et al., 2016; Filippone et al., 2020; Legros et al., 2020). More recently, ZIKV replication, lesions, and atrophy were observed in the skeletal muscles of an animal model of ZIKV infection (Gavino et al., 2021; Hirsch et al., 2017; Filippone et al., 2020). Using this animal model, it was shown that ZIKV replication in skeletal muscle precedes virus detection in the brain (Gavino et al., 2021). Here, we demonstrated that ZIKV replicates in myoblasts, proliferative progenitor muscle cells, characterized by the release of infectious viral particles into the culture supernatant, and the viral envelope protein is detected in approximately 50% of the myoblast population in culture. However, myotubes, already differentiated and fused myoblasts, are resistant to the infection (data not shown), confirming our previous results (Legros et al., 2020; Riederer et al., 2022). Indeed, although ZIKV can enter myotubes, there is no progress in genome replication within these multinucleated cells, in which several antiviral genes are enhanced (Riederer et al., 2022). In contrast to our findings, Gavino-Leopoldino et al. ; (Gavino-Leopoldino et al., 2021) detected myotube infection by ZIKV, but using a MOI 50 times higher than what we applied in our protocol, confirming a much lower sensitivity of myotubes to infection as compared to myoblasts.

In agreement with our results, Jaquet and collaborators have shown that susceptibility to ZIKV infection depends on the stage of differentiation at which cells are most resistant (Jaquet et al., 2024). However, in myoblasts infected with a MOI of 1, a higher percentage of infected cells and cytopathic effects was observed at 72 hpi (data not shown); despite this MOI, myotubes remained resistant. These results suggest that increasing virus concentration *in vitro* will negatively impact the cellular responses. However, it is very challenging to determine the viral load in tissues and organs, as living organisms exhibit complex responses involving interactions between immune cells, extracellular matrix components, and other signaling pathways that are not present *in vitro* models. At an MOI of 0.1, infected myoblasts were viable, without exhibiting cytopathic effects, allowing for functional assays with minimal variables. On the other hand, half of the culture was positive for the virus, exhibiting productive infection, with myotubes still resistant. We consider that our model represents a window into the initial phase of an *in vivo* infection, where proliferating myoblasts become targets of ZIKV, which can consequently severely impact the muscle repair and regeneration process, while also contributing to the spread of the virus throughout the body.

Herein, we also evaluated the proliferation, adhesion, migration, and differentiation of human myoblasts infected with ZIKV, without conducting studies on the molecular mechanisms related to these functional assays, such as signaling pathways and protein expression, which will be explored in subsequent studies. These biological events are of fundamental importance for myogenesis during skeletal muscle growth, homeostasis/maintenance, repair, and regeneration (Taylor et al., 2022; Wu and Yue, 2024).

In cultures of ZIKV-infected myoblasts, the reduced number of cells was due to alteration in the cell cycle, which regulates DNA replication, cell growth, and cell division (Lemmens and Lindqvist, 2019). Interestingly, infected myoblasts (positive for the E viral envelope protein) were almost all EdU-negative, indicating that the presence of the virus disrupts the proliferation capacity, rather than because of a secreted molecule, which could affect the entire culture. Additionally, we observed a decrease in the percentage of the Ki-67^high^ population in ZIKV-infected myoblasts. As KI67 is absent in the G0 phase and its expression peaks during mitosis, our results suggest that progression to the S phase is being inhibited or delayed, resulting in fewer cells completing cell division. This finding is in agreement with the results, which show a reduction in the number of cell divisions (**Figure 1F**), and is supported by the absence of cell death (apoptosis and necrosis, **Figure 1H**) in infected cells. This suggests that the reduction in myoblasts in culture is due to disturbances in cell cycle arrest.

Indeed, analysis of the transcriptional profile of human myoblasts infected with ZIKV, through Reactome pathway annotations, revealed a negative enrichment of cell cycle-related pathways including “DNA damage,” “mitotic phase,” “G2-M checkpoints”, as well as downregulation of genes encoding several histones, and KEGG pathway annotations revealed a positive enrichment of p53 signaling, among antiapoptotic genes, respectively, in ZIKV-infected myoblasts vs MOCK myoblasts (The RNA-seq data dataset used in our study is publicly available in SRA-NCBI (www.ncbi.nlm.nih.gov/sra), SRA accession PRJNA662490. The RNA-seq data are available on the following websites: http://biotools.labinfo.lncc.br/muscle_zika/forgeneexpression). Moreover, ZIKV can interfere with and delay apoptosis, promoting virus replication by preventing premature cell death and creating a sustained environment for the virus (Turpin et al., 2019). ZIKV NS5 protein dysregulates the mitotic spindle and induces DNA damage by modulating cell cycle checkpoints and p21 and p53 expression, thereby facilitating the synthesis of more viral proteins to enhance viral replication (Lebeau et al., 2024). These mechanisms explain ZIKV tropism for progenitor cells as well as its ability to inhibit the development of different tissues by disrupting key cellular processes in neural progenitor cells, including decreased survival, insufficient proliferation, DNA damage, and mitotic deregulation (Liang et al., 2016; Hammack et al., 2019; Vota et al., 2021; Rychlowska et al., 2022)

Adhesion is a fundamental process before cell migration, as it establishes the necessary interactions between cells and their surrounding environment, enabling subsequent movement. Perturbation of cell adhesion leads to impaired muscle growth and regeneration (Taylor et al., 2022). We also showed that myoblasts from ZIKV-infected cultures exhibited reduced adhesion capacity, with adhered cells being smaller and less spread, regardless of the presence of ZIKV within the cell, likely due to a bystander effect. In this sense, cells not directly infected by the virus end up being indirectly affected by factors produced by infected cells, such as cytokines or vesicles containing viral RNA or proteins. 502 Myoblasts and differentiated myocytes can migrate from regions distant from the injury site, complementing the number of mononucleated muscle cells necessary for repairing or regenerating damaged fiber (Hindi and Millay, 2022). Migration is also essential for myocytes to align correctly for fusion, facilitating cell adhesion (Kim et al., 2015). Confirming data from our group (González et al., 2017), LM-111 significantly enhanced myoblast migration compared to the condition without coating in the mock group, indicating that these cells can respond to a stimulus and improve their migratory capacity. However, in the ZIKV-infected cultures, human myoblast migration was strongly impaired. This is in keeping with the fact that in progenitor neural cells and trophoblasts, ZIKV infection also inhibits cell migration and disrupts the development of these tissues (Vota et al., 2021; Ohki et al., 2024). The transcriptional profile in ZIKV-infected myoblasts revealed GO-positive enrichment of pathways related to antiviral response, innate immune response, and cytokine production and signaling (Riederer et al., 2022). Analyzing this transcriptomics, we observed an upregulation of IFNI, IL1, IL6, IL12, CXCL12, CCL5, and ISGs (IFN-stimulated genes, while Reactome analysis showed downregulation of pathways related to NOTCH, FGFR, and ECM in ZIKV-infected myoblast cultures (The RNA-seq data dataset used in our study is publicly available in SRA-NCBI (www.ncbi.nlm.nih.gov/sra), SRA accession PRJNA662490. The RNA-seq data are available on the following websites: http://biotools.labinfo.lncc.br/muscle_zika/forgeneexpression). All of these genes and pathways are involved in cell proliferation, adhesion, and migration and can affect the entire culture, including non-infected cells (Xie et al., 2020; Karamanos et al., 2021; Gioftsidi et al., 2022; Xue et al., 2025).

**Figure 6:**
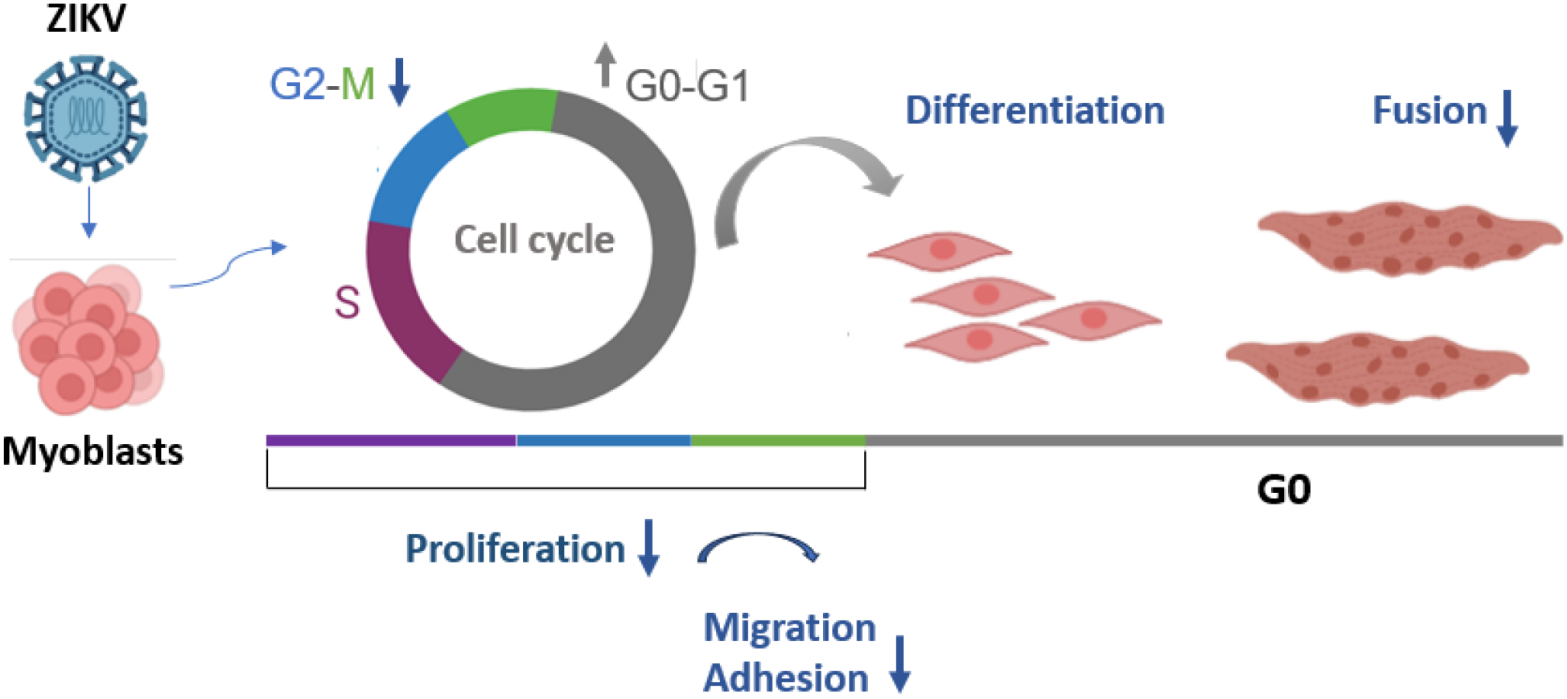
ZIKV infection affects muscle myogenesis *in vitro*: Myoblast infection by ZIKV impairs cell cycle progression, preventing cell proliferation, and disrupting adhesion, migration, and myoblast fusion, key biological processes essential for skeletal muscle regeneration.

The formation and regeneration of skeletal muscles rely on the fusion of activated muscular progenitor cells, myoblasts, to form multinucleated syncytia (called myotubes *in vitro*) that occurs in a series of steps, including cell migration, adhesion, and signaling transduction pathways (Schmidt et al., 2019). ZIKV infection strongly inhibited myotube differentiation from infected myoblasts, and this was due to the presence of ZIKV, as a viral replication inhibitor reverted the fusion capacity to a level similar to that of the MOCK controls, including the number of nuclei. When we infected myoblasts at high confluence and induced differentiation immediately after exposure to ZIKV, myotube formation was also impaired, but to a lesser extent than the poorly differentiated 3-day-infected myoblasts. This result suggests that the longer the exposure time to the virus, the greater the damage will be, with consequent reduction in cell adhesion, migration, and fusion capacity. Interestingly, after 3 days of differentiation from infected myoblasts, we observed that most infected cells were myocytes, with few ZIKV E protein-positive myotubes, suggesting that fusion and myotube formation are particularly affected. In support of our findings, the transcriptional profile analysis in ZIKV-infected myoblasts showed downregulation of pathways related to “muscle development”, “cell differentiation”, and “myoblast fusion”, among others, and also the downregulation of the MyoD (MYOD1) and Myogenin (MYOG) genes, with log2 fold changes of -1.6 and -2.0, respectively (The RNA-seq data dataset used in our study is publicly available in SRA-NCBI (www.ncbi.nlm.nih.gov/sra), SRA accession PRJNA662490. The RNA-seq data are available on the following websites: http://biotools.labinfo.lncc.br/muscle_zika/forgeneexpression). On a systemic level, whether muscle can contribute to viral dissemination to other tissues or long-term effects are unknown. A question remains as to why ZIKV replicates only in undifferentiated human muscle cells, whereas it replicates in mouse skeletal muscle, promoting severe damage (Aliota et al., 2016; Gavino et al., 2021). We may speculate that because these authors used a mouse model of ZIKV maternal transmission, they were able to observe necrosis, inflammatory foci, and fiber atrophy in fetuses and newborns, periods of life during which proliferative myoblasts are present, making them permissive to virus infection. Consistent with these results, myoblasts are readily permissive to ZIKV infection in our *in vitro* model. On the other hand, we found that differentiated and fused myoblasts (myotubes) are resistant to ZIKV infection, a finding compatible with the mild symptoms observed in adult ZIKV-infected subjects. Overall, our results suggest that ZIKV infection affects myogenesis *in vitro* by modulating key steps in myogenesis and regeneration, including proliferation, migration, adhesion, and differentiation/fusion (**Figure 6**). The absence of interaction with molecules and cells that make up the architecture of normal tissue is a significant limitation of this study. Co-culture systems or organoids, which incorporate immune cells and ECM components, will expand the discussion to consider potential *in vivo* consequences. This work presents novel findings critical to understanding the pathology of ZIKV in skeletal muscle and has implications for the search for new strategies to prevent and treat viral infections affecting the skeletal muscle system.

## Data Availability

The raw data supporting the conclusions of this article will be made available by the authors, without undue reservation.
