# Supplementary material for: Zika virus infection disturbs development of human muscle progenitor cells
Source: Front Cell Infect Microbiol. 2026 Jan 30;15:1638589. doi: 10.3389/fcimb.2025.1638589 (PMC12901458; doi:10.3389/fcimb.2025.1638589)

## Supplementary Material

**Supplemental Figure 1: Flow cytometry gating strategy for myoblast analysis using FlowJo. (A)** A time gate was applied to exclude artifacts, **(B)** followed by a singlet gate that excluded doublets (FSC and FC-A). **(C)** Gate of myoblast population based on forward (FSC) and side scatter (SSC) to include the cell area of interest. **(D)** Representative gate of live cells using FVS 520 viability in MOCK cells. **(E)** Gate of myoblasts positive to 4G2 (E-protein flavivirus marker) in ZIKV-infected cells, and **(F)** ki67+ using anti-ki67 BV421in ZIKV-infected cells.

**Supplemental Figure 2: Flow cytometry gating strategy for myoblast cell cycle analysis using FlowJo**. **(A)** For the cell cycle analysis of ZIKV and MOCK myoblasts, a time gate was applied to exclude artifacts, **(B)** followed by a singlet gate excluding doublets (PI-A and PI-W). **(C)** Gate of myoblast population based on forward (FSC) and side scatter (SSC) to include the cell area of interest, and **(D)** histogram of PI-A population in linear scale.

**Supplemental Figure 3: Flow cytometry gating strategy for myoblast apoptosis analysis using FlowJo**. For the apoptosis analysis performed by cytometry flow of ZIKV and MOCK myoblasts 72 hpi **(A)** a time gate was applied to exclude artifacts, followed by **(B)** a singlet gate excluding doublets (FSC-A and FSC-H). **(C)** Gate of myoblast population based on forward (FSC) and side scatter (SSC). **(D)** Representative experiment analyzed using the Annexin V-FITC versus PI scatter plot to distinguish viable, apoptotic, and necrotic cells at 72 hpi.

**Supplemental Figure 4: ZIKV infection reduces myoblast adhesion capacity on LM-111. (A)** A representative scheme of adhesion assay. At 72 hpi (MOI 0.1), ZIKV and MOCK cultures were trypsinized and added to glass coverslips pre-coated with LM-111 to adhere for 2 h. Adherent myoblasts identified by immunofluorescence were quantified and phenotyped using ImageJ software. The scheme was created using BioRender (BioRender.com). **(B)** Representative image of adhered cells stained for desmin (green), E viral protein (red), and nuclei (DAPI). The lower panels are higher-magnification views of the upper panels. Scale bar 100 µm. The bar graph shows the mean ± SD of the number of adhered ZIKV and MOCK myoblasts per field (ten to fifteen fields per coverslip).

Statistical differences *p<0.05 using Unpaired T-test. **(C)** The bar graph shows the mean ± SD of the cell area and roundness of ZIKV and MOCK myoblasts per field. Statistical differences *p<0.05 using Unpaired t test with Welch's correction. Representative data from two independent experiments in triplicate. **(D)** Pearson Correlation shows no significant correlation between percentage of infection and cell area in ZIKV-infected myoblast cultures (r=0.01, p<0.691). The y-axis shows the percentage of infected myoblasts (4G2+), and the x-axis shows the average cell area per well. Representative data are expressed as mean ± SD of two independent experiments in duplicates.

**Supplemental 5: NITD008 ZIKV-inhibitor cytotoxicity assay.** Myoblasts were infected with ZIKV for 2 h (MOI 0.1), followed by treatment with the viral replication inhibitor NITD008 at concentrations of 5 µM, 2.25 µM, and 1.12 µM in PM. After 72 hours post-infection (hpi), supernatants were collected, and the viral titer was quantified in VERO cell monolayers using the plaque-forming unit (PFU) assay. **(A)** Representative images showing the plaque morphology of ZIKV-infected myoblast cultures, with different NITD008 concentrations described above. **(B)** The Bar graph shows the antiviral activity of the NITD008. **(C)** Dot plot representative of infected myoblasts treated with NITD008 (green) and negative control of treatment with vehicle (red), MOCK (blue), and blank control (gray), analyzed by cytometry flow. **(D)** Cell cytotoxicity was assessed by measuring LHD activity in the culture supernatants of ZIKV and MOCK myoblasts with NIT008 and DMSO as a treatment control. One representative experiment of viral replication inhibition in myoblasts.

**Supplemental 6: ZIKV reduces the fusion of infected-confluent myoblast cultures. (A)** A representative scheme of differentiation assay. Myoblasts at high confluence were infected with ZIKV (MOI 0.1), and after 2 h of incubation, cells were induced to differentiate for 3 days. **(B)** Representative images of immunofluorescence microscopy show myotubes formed from ZIKV and MOCK-infected myoblasts, along with the total number of nuclei. Differentiated cells positive for MyHC (red) and nuclei with DAPI (blue). Scale bar 100 µm. **(C)** The bar graph shows the mean ± SD of the fusion capacity in ZIKV and MOCK cells. The fusion index was calculated by the ratio of the number of nuclei within fused myotubes (MyHC+) cells containing 2 or more nuclei versus the total number of nuclei. Statistical differences **p<0.01 using Unpaired T-Test. ns: no statistical difference. **(D)** The bar graph shows the total nuclei number in ZIKV and

MOCK cells. Ns: no statistical difference. **(E)** The graphic shows the average number of myocytes per field. Myocytes were defined as differentiated mononuclear cells (MF20+). Statistical differences *$p<0.05$ using Unpaired T-Test. Representative data are expressed as mean ± SD of two independent experiments in triplicate.

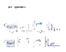

# Supplemental figure 1

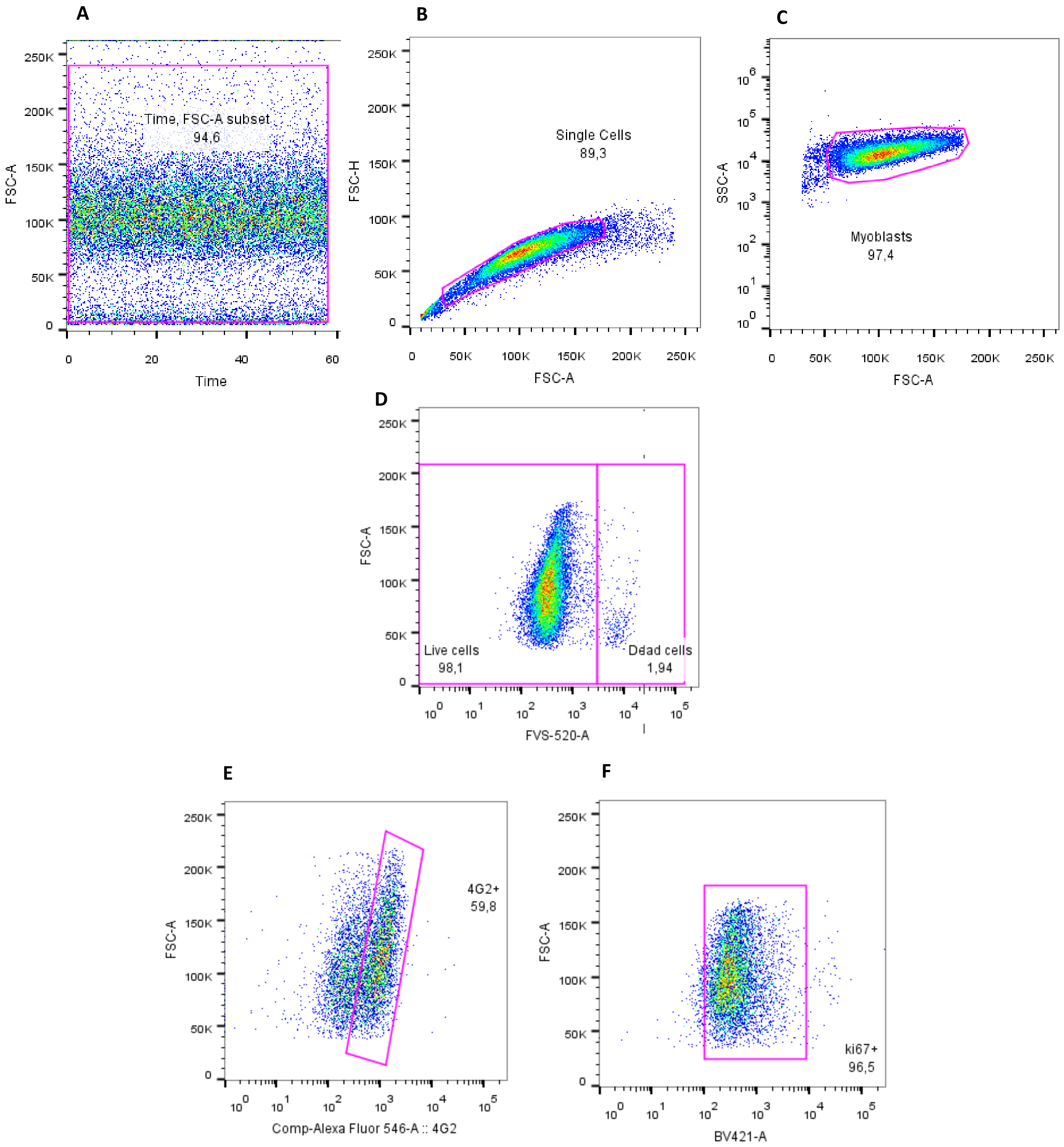

# Supplemental figure 2

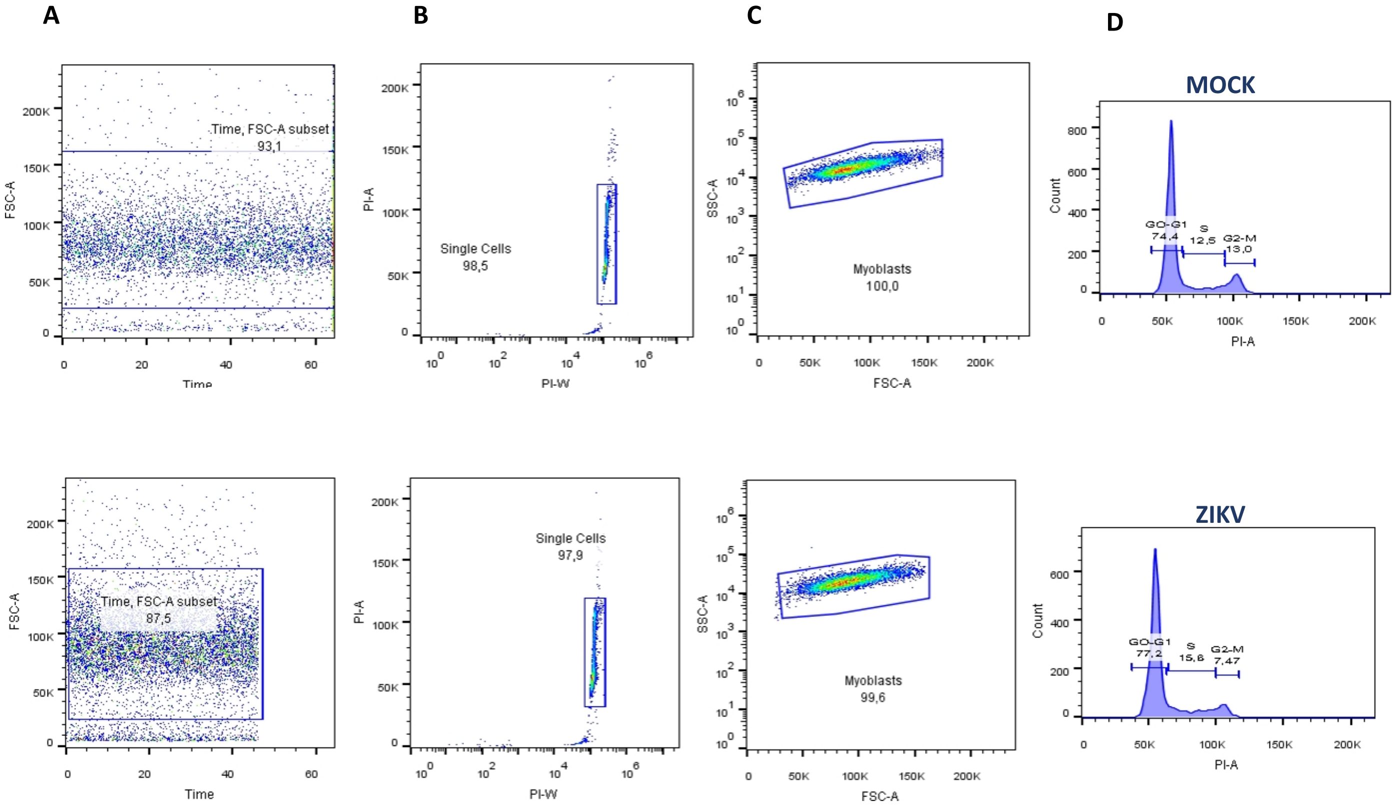

# Supplemental figure 3

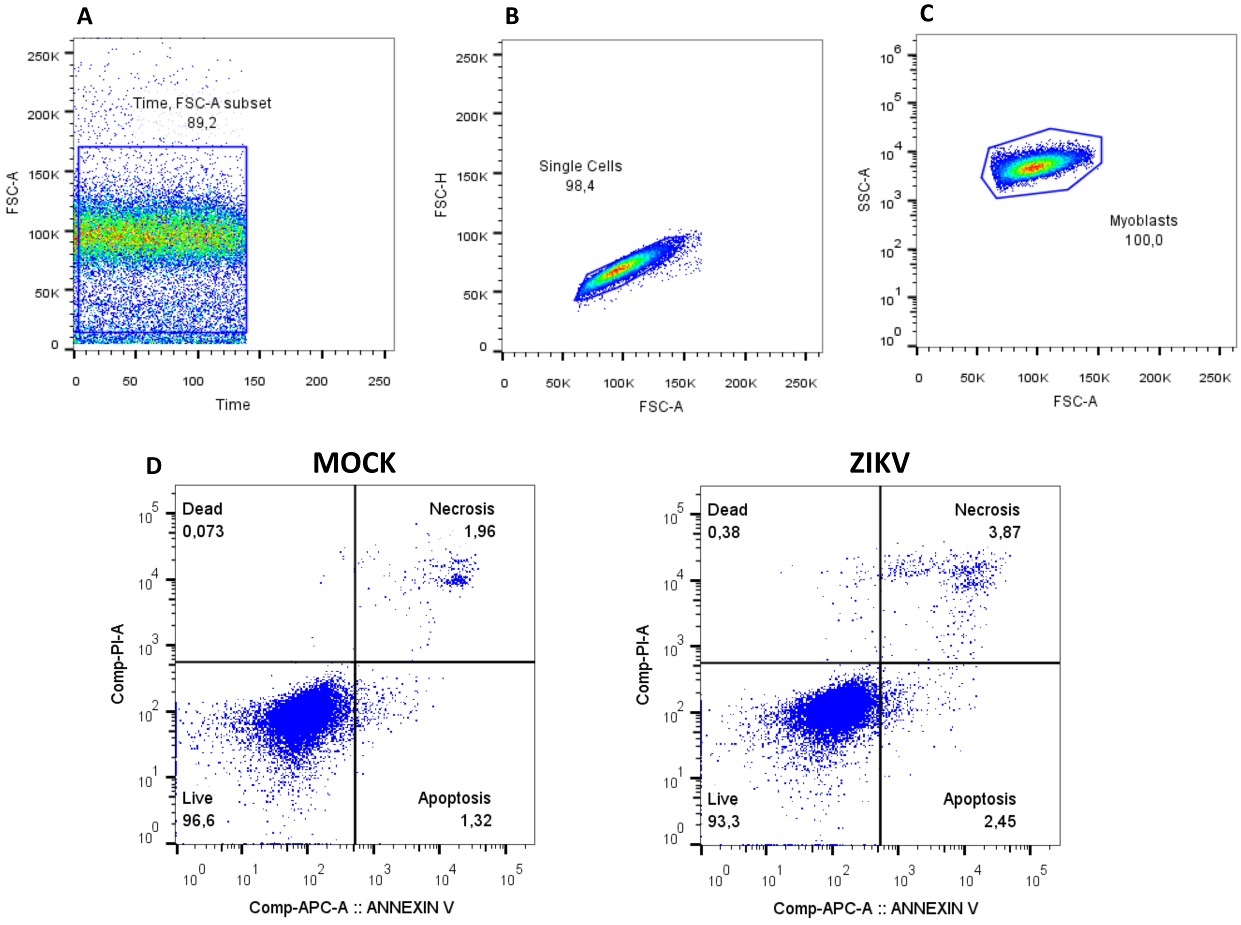

# Supplemental figure 4

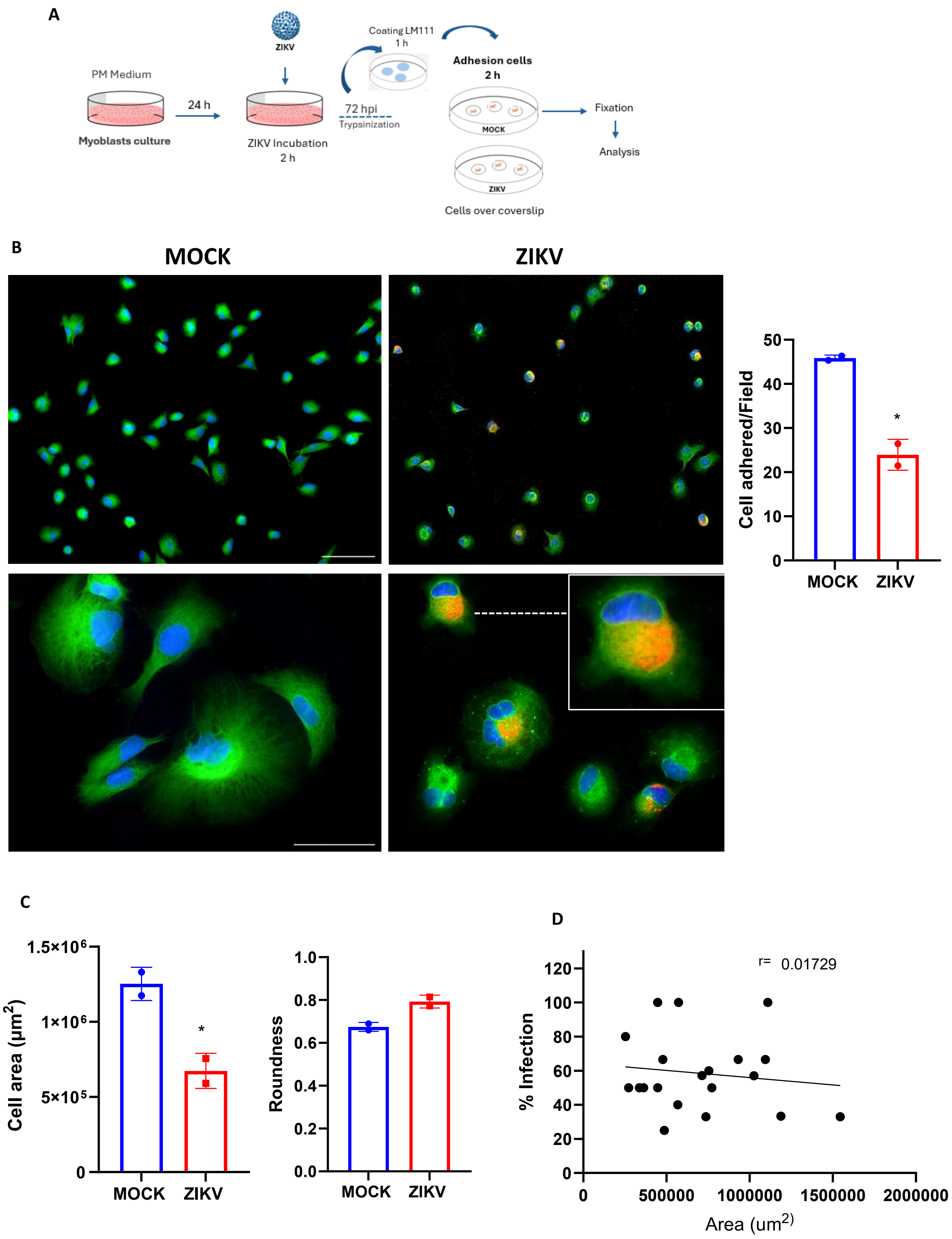

# Supplemental figure 5

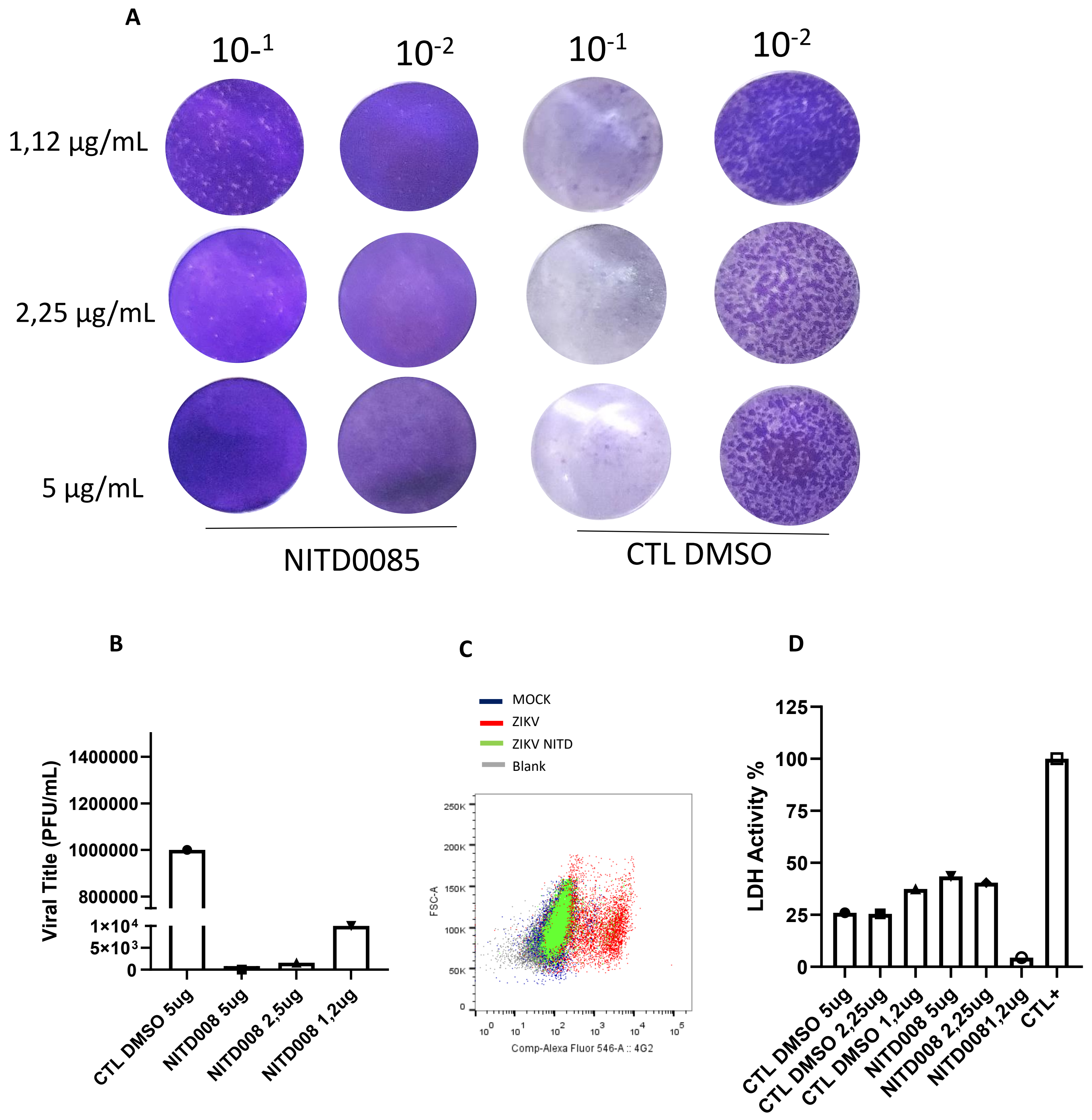

# Supplemental figure 6

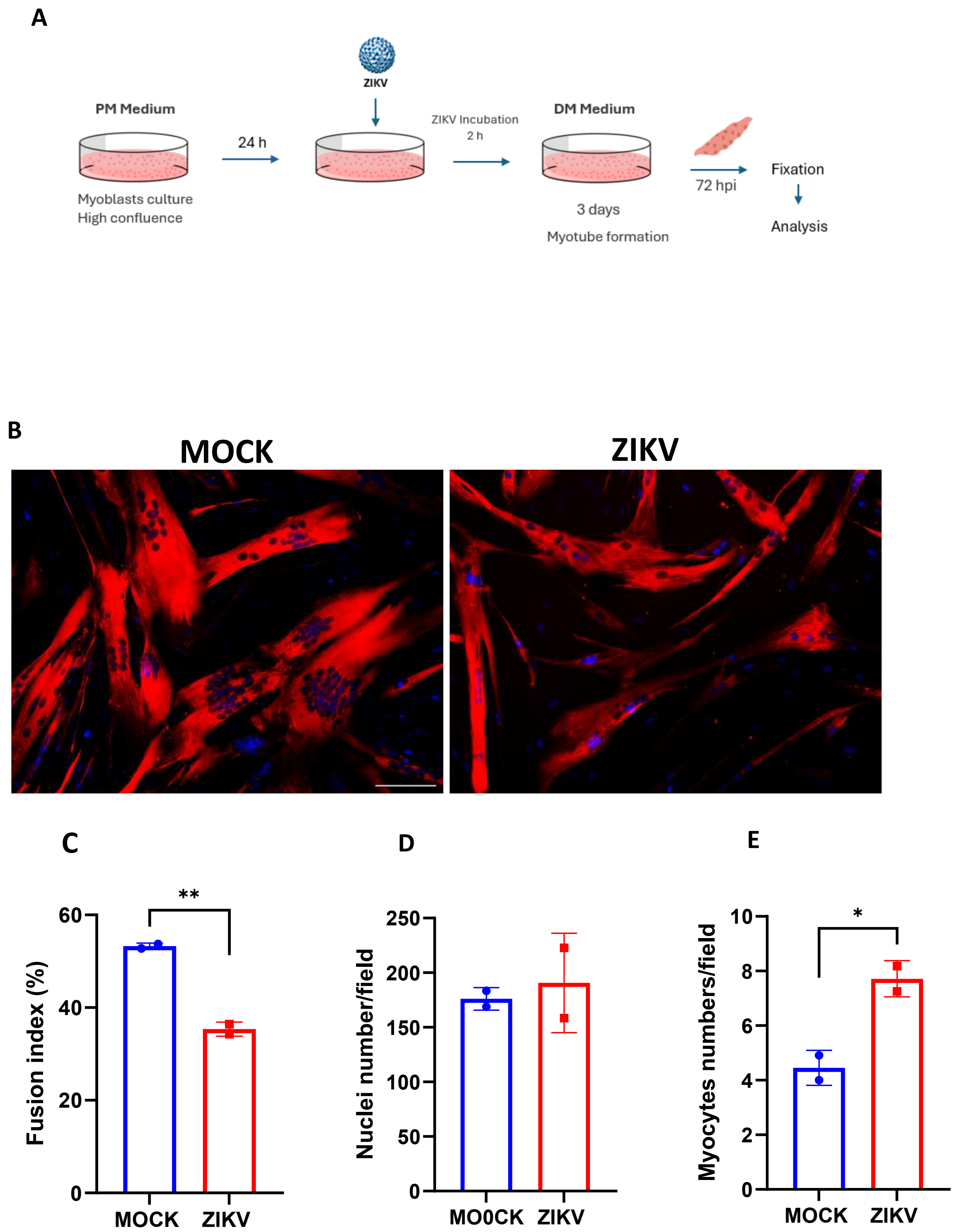

Supplement: Supplementary file 1 [file DataSheet1.pdf]
